# Prosodic regularity and reader reception in three-word rhyming couplets: a four-pronged investigation of *Dizi Gui*

**DOI:** 10.3389/fpsyg.2026.1904387

**Published:** 2026-09-30

**Authors:** Zheng Wang, Sheng Yu

**Affiliations:** School of the English Language & Culture, Xiamen University Tan Kah Kee College, Zhangzhou, China

**Keywords:** *Dizi Gui*, lexical concentration, perceived mnemonic effectiveness, prosodic regularity, reader reception, three-word rhyming couplets

## Abstract

Prosodic regularity—characterized by fixed rhythm, end-rhyme, and syntactic parallelism—has been theorized to enhance memorability, but there is limited empirical evidence linking these features to cognitive mechanisms. This study examines whether textual characteristics associated with prosodic regularity and reader-perception patterns are consistent with theoretical accounts of reduced processing demands and chunking-based mnemonic support. Using a corpus-based four-pronged framework, we compared four English translations of a canonical classical Chinese text *Dizi Gui*, a three-word rhyming couplet version (high prosodic regularity) and three control varieties. Results showed that the rhyming couplets exhibit extreme syntactic compression (mean length = 6.32 words), high lexical diversity (STTR = 47), and a Zipfian slope (−0.65) mirroring the ST (−0.66), indicating a similar macro-level pattern of lexical concentration. Reader data showed that the three-word rhyming version with supplementary annotation received the highest proportions of selections for perceived mnemonic effectiveness (64.35%), aesthetic appeal (42.61%), and popularization potential (57.39%). These converging findings suggest that prosodic regularity may reduce processing demands and support working-memory consolidation through chunking mechanisms, while direct experimental tests of these cognitive mechanisms remain necessary.

## 1 Introduction

Human language comprehension involves not only semantic and syntactic processing but also the extraction of prosodic information—rhythm, intonation, and stress patterns—which facilitates memory encoding and reduces processing effort (Cutler et al., 1997; Frazier et al., 2006; Wagner and Watson, 2010). In particular, prosodic regularity, characterized by predictable rhythmic and melodic patterns, has been shown to enhance working memory consolidation through chunking mechanisms (Miller, 1956; Thalmann et al., 2019; Zora et al., 2023; O’Leary et al., 2025) and reduce cognitive load during sentence processing (LaCroix and Ratiu, 2025). This effect, rooted in Cognitive Load Theory (CLT), operates on a fundamental principle: human working memory has a limited capacity, and any reduction in structural parsing effort frees attentional resources for information retention. In settings of highly regular prosodic structure, as in poetry, rhyming couplets, or chanted verses, the brain can allocate fewer resources to structural parsing and more to information retention (Blain et al., 2022; Gkintoni et al., 2024). Importantly, written presentation can mitigate the transient-information burden on working memory when verbal material is lengthy because written information is more permanent than auditory input (Leahy and Sweller, 2011). At the same time, skilled readers process prosodic syllable information early in visual word recognition and activate elaborated, speechlike phonological representations during silent reading (Ashby and Martin, 2008). Conceptually, visual permanence preserves access to information, whereas prosodic regularity may provide an additional organizational benefit by supplying predictable structural cues for chunking and retention. This distinction makes it theoretically important to examine whether the observed textual and reader-perception patterns are compatible with such a theoretically proposed cognitive benefit in visually presented texts, which motivates this study.

Despite this potential cognitive advantage, most existing research on prosodic processing has traditionally focused on auditorily presented materials in controlled laboratory settings (Cutler et al., 1997; Pedersen et al., 2000; Frazier et al., 2006; Ceravolo et al., 2016). Much less is known about how prosodic regularity affects comprehension, memorization, and cognitive load in naturalistic, visually presented texts that readers encounter in everyday contexts, such as instructional texts, poetry, or translated works. This gap is particularly significant for vocative texts (texts intended to instruct, persuade, or influence behavior), where memorability and audience engagement are central to cross-cultural communicative success (Trosborg, 1997). Driven by this expanding need for cross-cultural communication, there has been a burgeoning global interest in Chinese language pedagogy (Gong et al., 2020) and a worldwide dissemination of classical Chinese philosophy, thrusting traditional texts for moral instruction into the international spotlight. For non-native readers of these moral-instructional texts, however, comprehending and retaining such culturally dense philosophical concepts frequently poses a formidable cognitive bottleneck. This bottleneck, as recent evidence suggests (Jiang et al., 2026; Qiu et al., 2026), arises because the unadapted, highly elliptical structures of classical Chinese impose a heavy intrinsic cognitive load that rapidly exhausts the reader’s finite working memory resources. More importantly, the cognitive mechanisms underlying highly compressed and prosodically constrained visual genres, such as three-word rhyming couplets, remain underexplored. In such texts, syntactic ellipsis and rhythmic symmetry often substitute for explicit grammatical structure, raising important questions about how readers process, retain, and evaluate information presented in highly condensed forms. Addressing these questions requires a methodological framework capable of linking textual structure to cognitive outcomes, a need that brings us to the intersection of corpus-based translation studies (CBTS) and cognitive load theory.

Pioneered by Baker (1993, 1999) and further developed by scholars such as Laviosa (2004), CBTS has extensively documented formal variation across translation styles, with literary translation remaining a major area of inquiry (Murphy, 2015; Zanettin et al., 2015; Granger and Lefer, 2022). Recent studies have applied corpus methods to compare translations of classical Chinese texts, quantifying differences in lexical diversity, sentence length, and phraseological patterns (Liu and Afzaal, 2021; Meng and Pan, 2022; Wang, 2023; Chou and Liu, 2024). This body of research, however, has predominantly focused on identifying and describing stylistic variation through corpus-based quantitative analyses, while comparatively limited attention has been paid to the potential cognitive implications of such variation for readers. As a result, longstanding debates concerning the relationship between form preservation and meaning transmission in translation continue to rely largely on textual and theoretical evidence rather than empirical evidence concerning reader cognition (Halverson, 2010; Saldanha, 2011).

This gap reflects two interconnected methodological challenges: the lack of semantically controlled text materials exhibiting systematic variation in prosodic regularity, and the absence of an integrative framework capable of linking textual structure, information-theoretic properties, and reader responses. Classical Chinese texts, characterized by highly regular patterns of rhythm, rhyme, and parallelism that were traditionally designed to facilitate memorization and oral transmission (Kern, 2010), provide an especially suitable context in which these issues can be investigated. Among such texts, *Dizi Gui* (Chinese characters “弟子规”; *Dizi* means “disciples” or “students” and *Gui* means “Standards”)—compiled during the Qing dynasty and composed of 360 trisyllabic lines featuring syntactic parallelism (see Figure 1 for an excerpt)—was explicitly designed for oral recitation and memorization.

**Figure 1:**
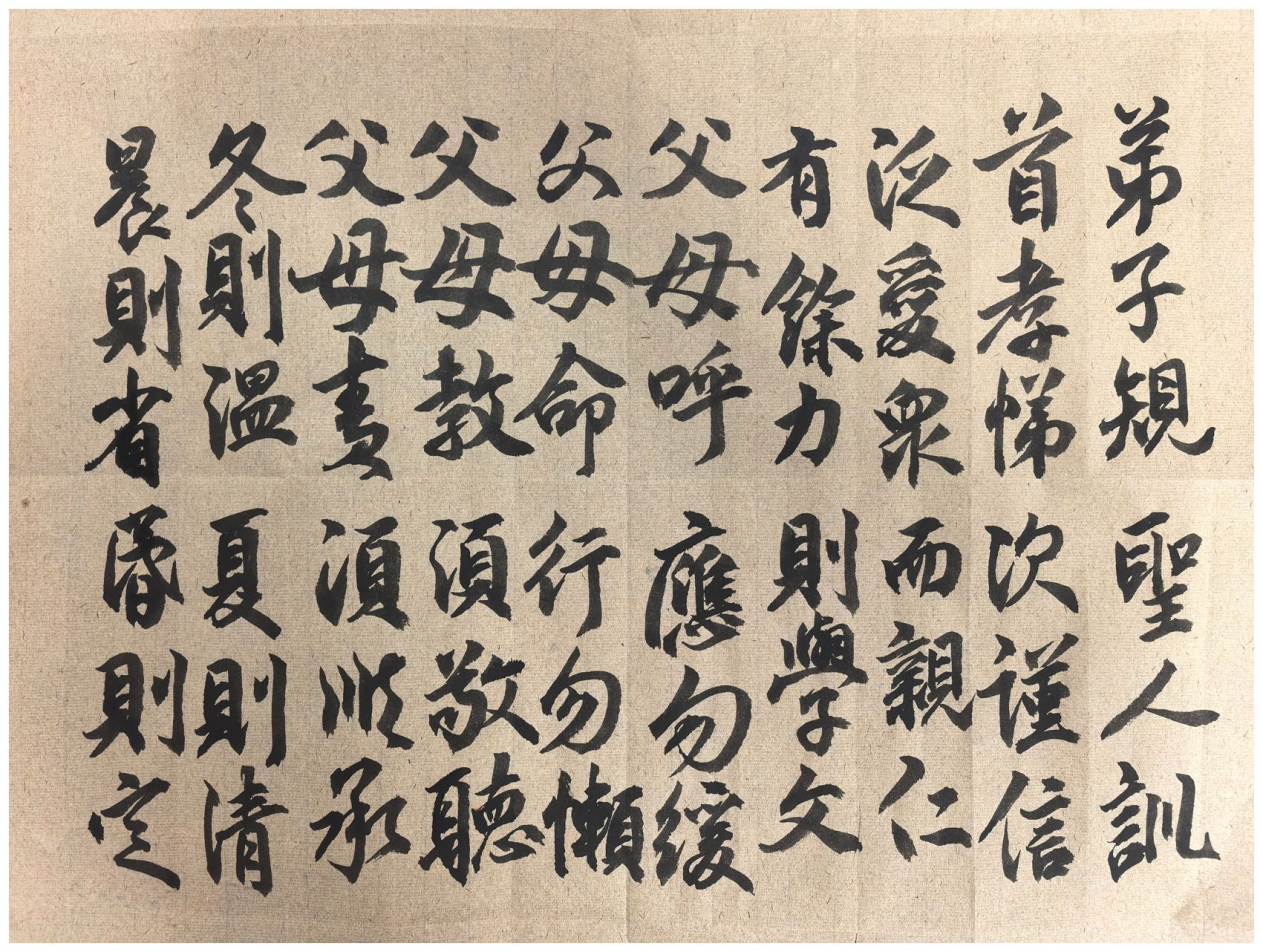
*Dizi Gui* in classic Chinese (an excerpt) (Source: created by the authors).

Four of its existing English translations form a natural, semantically matched continuum of prosodic regularity. Zhao’s (2018) *Canons for Disciples: In English Rhyme* (*CFD*), the highest-prosodic-regularity version, comprises 360 three-word rhyming verses and their corresponding prose annotation; each verse line contains exactly three words, with contractions counted as single words to preserve the metrical constraint (Figure 2). Moving from highest to lowest prosodic regularity are Gu’s (2010) poem-style translation, Pure Land Learning College (2005) interpretive *Dizi Gui: Guide to a Happy Life*, and *CFD*’s prose annotation, which progressively prioritize semantic explanation over prosodic structure.

**Figure 2:**
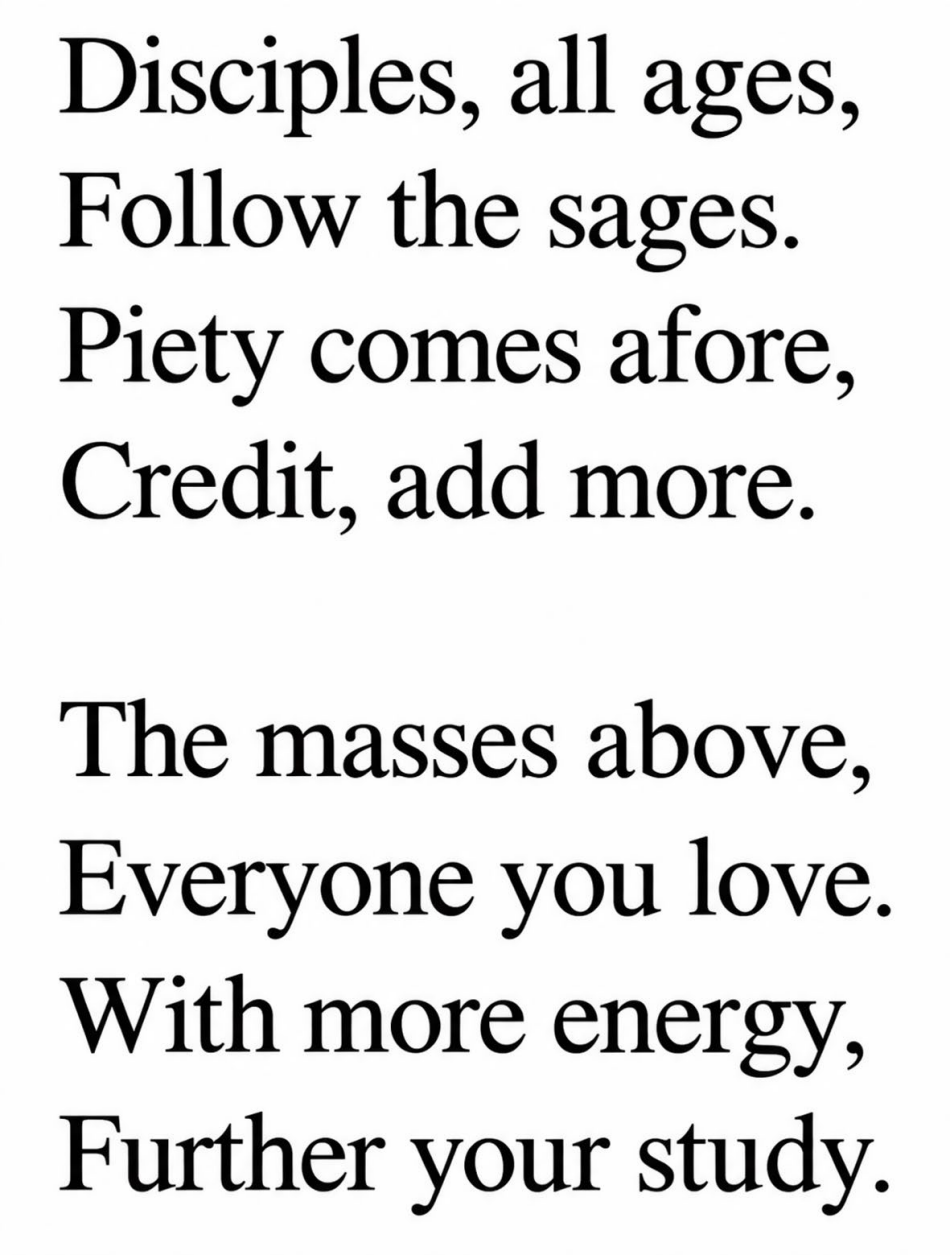
Excerpt from Zhao’s (2018) three-word translation (text reproduced verbatim; figure layout prepared by the author).

To address these challenges, the present study adopts a four-pronged converging framework integrating (1) corpus analysis to quantify lexical and syntactic features, (2) Zipfian distribution modeling to characterize macro-level lexical concentration, (3) qualitative textual analysis to examine specific prosodic strategies, and (4) reader perception analysis to evaluate reader preferences across perceived mnemonic effectiveness, aesthetic appeal, and popularization potential. By combining evidence from textual structure, information-theoretic patterns, and reader responses, this framework provides a more comprehensive perspective on how prosodic regularity may influence cognitive processing in translated texts.

Against this backdrop, the present study investigates how prosodic regularity may influence cognitive processing and whether it is associated with stronger perceived mnemonic effectiveness in visually presented three-word rhyming couplets, using *Dizi Gui* and its four English translations as a case study. Specifically, the study addresses the following research questions (RQs):

*RQ1*: What are the fundamental lexical and syntactic distinctions between the three-word rhyming couplets (high prosodic regularity) and other text varieties (lower prosodic regularity)?

*RQ2*: Does prosodic regularity produce a pattern of lexical concentration (as indexed by Zipfian slopes) similar to that of the source text (ST)?

*RQ3*: How do readers evaluate the three-word rhyming couplets relative to other text varieties in terms of perceived mnemonic effectiveness, aesthetic appeal, and popularization potential, and how might their prosodic, syntactic, and presentation features be associated with these preferences?

## 2 Methodology

### 2.1 A four-pronged analytical framework

Following recent calls for methodological pluralism in text studies (Pérez-Paredes and Curry, 2024), we operationalized a multi-method framework to capture the linguistic, structural, and perceptual dimensions of text analysis. First, we used corpus analysis to quantify lexical and syntactic patterns, including entropy (Baayen, 2001), as indicators of communicative style. Second, Zipfian distribution analysis (Zipf, 1949) characterized lexical concentration through visual plots to reveal genre-specific deviations in repetition (Wang and Liu, 2023). Third, we adopted qualitative analysis to examine how syntactic and prosodic patterns may contribute to mnemonic and aesthetic functions via creative strategies. Fourth, reader perception analysis (Leder et al., 2004) grounded these structural descriptions in audience evaluations of perceived mnemonic effectiveness, aesthetic appeal, and popularization potential (White, 2004).

The following subsections will elaborate on the corpus analysis, reader perception analysis, and their accompanying statistical methods. Qualitative textual analysis will be presented in the Results section as a supplement to the quantitative findings.

### 2.2 Text data collection, processing, and analysis

From our institutional e-library we downloaded the PDF versions of Zhao’s (2018) three-word rhyming translation (with annotation), Pure Land Learning College (2005) interpretive translation, and Gu’s (2010) poem-style translation. These documents were converted to editable format using Adobe Acrobat Pro 2023 and manually verified for accuracy. For entropy analysis, we separated Zhao’s translation and its annotation into Word documents, as were the interpretive and poem-style versions. The four corpora were then analyzed using AntConc 4.2.0 (Anthony, 2022) and compared against the Brown and Frown reference corpora, two large, genre-diverse corpora widely used in corpus linguistics (de Dios, 2013). We adopted a multi-level analytical approach to capture lexical, syntactic, and information-theoretic features of the translations, as outlined below.

First, we calculated basic lexical and syntactic metrics to quantify overall stylistic differences across the four corpora. These included: (1) Average word length and proportion of small words (≤5 letters), to measure lexical simplification patterns; (2) Type-token ratio (TTR), to assess lexical diversity; (3) Average sentence length, to evaluate syntactic compression; (4) Repetition rate (RR), *P₁* (frequency of the most common word), and *Pcum₁₀* (cumulative frequency of the top 10 most frequent words), to quantify lexical repetition and concentration; and (5) Lexical uniqueness, defined as the percentage of word types that appear only once in the corpus, to measure lexical creativity and information density.

Second, we calculated Shannon entropy to measure information dispersion and linguistic heterogeneity across the four versions (Shannon, 1948; Altmann and Köhler, 2015). Entropy quantifies the uncertainty associated with predicting the next linguistic unit in a text, with lower values indicating greater textual monotony and higher values indicating greater information density. We computed both stratified entropy for words grouped by length (1 to 10 letters) (Length-Conditional Entropy, *H_L_*) and global text entropy for each corpus (*H_global_*) according to the following Equations 1 and 2:

$$H(W|L)=-\sum_{w\in W_{L}}p(w|L)\log_{2}p(w|L)$$ (1)

where *W_L_* represents the sub-corpus vocabulary consisting exclusively of word types that contain exactly *L* letters, and *p*(*w*|*L*) is the conditional probability of word type *w* within that specific length stratum.

$$H_{\text{global}}=-\sum_{i=1}^{V}p(w_{i})\log_{2}p(w_{i})$$ (2)

where *V* denotes the vocabulary size (total number of unique word types) and *p*(*wi*) represents the probability of occurrence for the *i*-th word type.

Third, we conducted lexical bundle and high-frequency word analysis to identify recurrent phraseological patterns and stylistic markers. We extracted the top 10 high-frequency words and top 10 two-word lexical bundles for each corpus, as these units reflect conventionalized usage patterns and reveal systematic differences in translator style and communicative orientation. To ensure comparability across the four text varieties of unequal total word counts (ranging from 1,138 to 2,909 tokens), we normalized all raw frequency data for high-frequency words and lexical bundles to occurrences per 1,000 words. This normalization procedure follows standard corpus linguistic practice (Biber et al., 1998; McEnery and Hardie, 2012) and allows for direct cross-corpora comparison of lexical and phraseological patterns while reducing potential confounding effects of text length.

Fourth, we performed syntactic pattern analysis to identify the dominant grammatical structures of each translation variety. Based on the high-frequency two-word lexical bundles identified in *CFD*, two coders independently coded recurrent syntactic patterns according to their structural configurations and grammatical functions. These patterns included imperative constructions, conditional clauses, and noun phrase structures, among others (Table 1). The coding procedure demonstrated high inter-coder reliability (Cohen’s *κ* = 0.87), and all disagreements were resolved through consultation with a linguistics expert. The coded frequencies were then compared to examine how syntactic choices were adapted to accommodate prosodic constraints.

**Table 1:** Syntactical structure and grammar pattern of the three-word rhyming translation.

Top syntactical structures and grammatical patterns	Frequency
Positive imperative sentence	43
A + i. + [adjective(s)] + [noun object]	21
You +[verb] + [noun object]	20
Negative imperative sentence Do not or Do not+[verb] + [noun object]	17
If/should conditional clause	14
What’s + [adjective(s)] or+[noun object] + [verb]	13
with+	10

Finally, we conducted Zipfian distribution modeling to examine macro-level lexical concentration patterns. For each corpus, we plotted word frequency against rank on a log–log scale and fitted a linear regression model to estimate the Zipfian slope. Steeper slopes indicate more formulaic, repetitive language use, while flatter slopes indicate greater lexical diversity (Lü et al., 2013). This analysis allowed us to directly compare macro-level lexical concentration patterns of the translations with those of the ST.

### 2.3 Questionnaire study

The questionnaire study was approved by the first author’s institution. To examine reader reception of the three-word rhyming translation as an effective vocative genre, we conducted a questionnaire study. Based on classic sampling criteria (confidence level = 95%, margin of error = 5%, and population proportion = 50%; Sudman, 1976), an online sample size calculator^1^ indicated a minimum required sample of 109. Eligible participants were junior and senior English majors enrolled in Chinese–English translation courses who had obtained a passing grade or above in the most recent end-of-term Chinese–English written translation examination. This criterion was used to establish a minimum baseline—rather than a direct assessment—of general Chinese source-text comprehension and Chinese–English translation ability, without specifically measuring classical-Chinese proficiency. Using convenience sampling (Salkind, 2022), we recruited 115 junior and senior English majors enrolled in Chinese–English translation courses at a regular 4-year university. They voluntarily participated after providing written informed consent. The sample included 82 females and 33 males, aged 19 to 22 years (*M* = 21.2, *SD* = 0.755). All participants were native Chinese speakers who had studied English from the third grade of primary school, classical Chinese from junior high school, and Chinese–English translation from their second year of university. By the time of this survey, they had received at least one and a half years of systematic training in translation theory, practical techniques, strategic decision-making, and cross-cultural communication. Prior familiarity with *Dizi Gui* was not assessed or used as a screening or stratification criterion. However, because all three translation conditions were based on the same ST, ST content was held constant across conditions.

Participants were given approximately 15 min to independently read each ST–translation pair before completing the evaluation questionnaire. This reading period was designed to allow sufficient time for participants to examine the linguistic features, stylistic characteristics, and semantic content of each translation without instructor guidance. For comparability across conditions, the same amount of reading time was allocated to all three materials: *CFD* (three-word rhyming translation with its annotation), poem-style translation (Gu, 2010), and interpretive translation (Pure Land Learning College, 2005). Importantly, in the *CFD* condition, the three-word rhyming text served as the primary reading material, with the prose annotation available as a supplementary aid, when needed, to clarify potential semantic ambiguities arising from its highly compressed form. Notably, the presentation order of the three translations was randomized across participants to reduce order effects (Schwarz, 1999).

After reading each translation, participants completed a questionnaire distributed via Wenjuanxing, in which they evaluated which translation style they perceived as having the highest mnemonic effectiveness, aesthetic appeal, and popularization potential (forced-choice format) (Brown, 2016). These three evaluation dimensions were selected based on the functionalist view of translation, particularly Reiss’s (2000) classification of text functions and Nord’s (1997) functional approach, which emphasizes that vocative texts are oriented toward eliciting responses from target readers. Accordingly, this study operationalized vocative effectiveness through three reception-oriented indicators: perceived mnemonic effectiveness, perceived aesthetic appeal, and perceived popularization potential. Perceived mnemonic effectiveness refers to participants’ judgment of how readily a translation can be retained and recalled, consistent with evidence that phonological patterning can facilitate recall and enhance mnemonic potential (Lindstromberg and Boers, 2008). Perceived aesthetic appeal refers to participants’ positive aesthetic judgment of a translation’s linguistic and formal qualities, drawing on Leder et al.’s (2004) information-processing model of aesthetic appreciation and judgment. Perceived popularization potential refers to participants’ judgment of a translation’s suitability for wider dissemination and acceptance among target readers, consistent with the receiver- and purpose-oriented principles of functionalist translation theory (Nord, 1997). These dimensions capture participants’ perceptions of whether a translation can attract readers’ attention, facilitate retention, and support wider acceptance among the target audience. Moreover, the forced-choice format was deliberately operationalized to simulate realistic reader text-selection behaviors and to directly address the comparative nature of the reader-reception question. Specifically, rather than eliciting independent absolute ratings of each translation, participants were required to identify the translation they perceived as most favorable on each evaluative dimension. This format therefore aligned the response task with the study’s focus on relative preference among competing translation styles. To ensure data quality, we screened questionnaire completion times using a minimum threshold of 30 s to identify potentially insufficient-effort responses, following recommendations in survey research (Huang et al., 2012; Ward and Meade, 2023). Because participants were given 15 min to read each translation before completing the questionnaire, this threshold applied only to the questionnaire response phase, which included demographic information and three forced-choice items. All 115 participants exceeded this threshold. Because the questionnaire required participants to select an answer before proceeding to the next item, the dataset contained no missing values. We therefore retained all responses for analysis.

### 2.4 Statistical analysis of questionnaire results

The questionnaire yielded nominal categorical data, as participants were required to select one translation style as having the highest perceived mnemonic effectiveness, aesthetic appeal, and popularization potential. Because the response variable consisted of frequency counts rather than continuous measurements, we employed non-parametric frequency-based statistical analyses, which are specifically designed to evaluate distributions of categorical preferences.

To test whether reader preferences deviated from an equal distribution across the three translation styles, we first conducted chi-square goodness-of-fit tests for each of the three evaluative dimensions. Under the null hypothesis of no systematic preference difference, each translation style was expected to receive an identical proportion of responses, corresponding to an expected frequency of approximately 38.33 responses per category for *N* = 115 participants. The chi-square statistic was calculated using the Equation 3:

$$\chi^{2}=\sum_{i=1}^{k}\frac{{\left(O_{i}-E_{i}\right)}^{2}}{E_{i}}$$ (3)

Where *Oi* represents the observed frequency of responses for category *i*, *Ei* represents the expected frequency under the null hypothesis, and *k* = 3 is the number of response categories. Degrees of freedom for all omnibus tests were calculated as *df* = *k* − 1 = 2. To identify which specific pairs of translation styles differed significantly when the overall test was significant, we performed *post-hoc* pairwise chi-square tests. To control for familywise Type I error inflation caused by multiple comparisons, we applied the Bonferroni correction method. Given that three pairwise comparisons were conducted for each dimension, we adjusted the conventional significance level of *α* = 0.05 to an adjusted threshold of *α* = 0.05/3 ≈ 0.017. Only pairwise comparisons with *p*-values below this adjusted threshold were considered statistically significant.

Furthermore, we calculated Cramér’s *V* as the standardized effect size for all omnibus chi-square tests, to quantify the magnitude of observed preference differences beyond mere statistical significance. Cramér’s *V* is a widely used and appropriate effect size measure for chi-square goodness-of-fit tests with three or more categories, as it ranges from 0 (no association) to 1 (perfect association) and is independent of sample size. Cramér’s *V* was calculated using Equation 4:

$$V=\sqrt{\frac{\chi^{2}}{N\times (k-1)}}$$ (4)

where *χ*^2^ is the chi-square statistic from the omnibus test, *N* is the total sample size, and *k* is the number of response categories. We interpreted effect sizes using Cohen’s (1988) conventional benchmarks: *V* = 0.10 indicates a small effect, *V* = 0.30 indicates a medium effect, and *V* = 0.50 indicates a large effect. For pairwise comparisons, we additionally calculated Cohen’s *h* (Equation 5) as a standardized effect size for the difference between two proportions (Cohen, 1988):

$$h=2\times \arcsin(\sqrt{p_{1}})-2\times \arcsin(\sqrt{p_{2}})$$ (5)

where $p_{1}$ and $p_{2}$ are the observed proportions for the two translation styles being compared. Cohen’s *h* was interpreted using the same conventional benchmarks: *h* = 0.20 indicates a small effect, *h* = 0.50 indicates a medium effect, and *h* = 0.80 indicates a large effect.

This analytical procedure was selected because it provides both a comprehensive global assessment of overall preference patterns and a precise examination of specific pairwise differences, while rigorously controlling for Type I error. By combining omnibus significance testing with standardized effect size estimation, we were able to draw robust conclusions about relative reader preferences across the three evaluative dimensions.

## 3 Results

### 3.1 Lexical and syntactic features of the four translation varieties (RQ1)

#### 3.1.1 Lexical features: word length and the proportion of small words

Table 2 shows the basic information for the four target text (TT) varieties and two reference corpora (Brown and Frown).

**Table 2:** Basic information for the TT varieties and reference corpora.

Corpus / TT variety	Word count	Average word length	Language
Brown	1,191,332	4.47	Modern American English
Frown	1,241,887	4.39	Modern American English
Three-word rhyming translation	1,138	4.13	An innovative rhyming form of English
The annotation	2,909	4.18	Oral/vernacular English language
Poem-style translation	2,495	4.27	Rhythmic poetic English
Interpretive translation	2,850	4.07	Prescriptive formal English

A distinctive formal feature of this instructional text emerges through lexical patterning. The average word length of the three-word rhyming translation is 4.13 letters. The proportion of small words (≤5 letters) is 65.03% in the rhyming translation and 67.17% in its annotation, while the Brown and Frown reference corpora exhibit lower small-word proportions at 63.94 and 63.71%, respectively. The high proportion of small words, together with an average word length of 4.13 letters, constitutes a genre-defining characteristic, reflecting a consistent pattern of lexical simplification that mirrors the conciseness inherent in the ST.

#### 3.1.2 Quantitative indicators of textual variation

Table 3 shows in detail the lexical data of our corpora and the two reference ones.

**Table 3:** Detailed lexical data of the TT varieties and reference corpora.

Measure	Brown	Frown	Three-word rhyming translation	English annotation	Poem-style translation	Interpretive translation
Tokens	1,191,332	1,241,887	1,138	2,909	2,495	2,850
Types	47,037	45,445	531	778	817	768
TTR	3.95	3.66	46.67	26.74	32.75	26.95
Sd. type/token ratio	44.68	45.79	47	37.05	44	37.49
Ave. word length	4.47	4.39	4.13	4.18	4.27	4.07
Sentences	42,564	56,925	180	188	183	187
Sd. Sent. length	22.88	15.3	6.32	15.47	13.63	15.24
1-letter word	38,603	44,264	71	87	51	265
2-letter word	171,837	209,927	138	505	429	603
3-letter word	302,087	374,288	225	677	569	419
4-letter word	249,219	162,730	306	685	552	604
5-letter word	110,469	113,649	162	259	258	274
6-letter word	85,903	89,201	100	306	285	237
7-letter word	77,518	82,558	73	178	160	212
8-letter word	56,189	59,775	38	95	96	85
9-letter word	39,867	43,002	18	61	44	68
10-letter word	26,897	28,450	7	56	51	83

‘Ave.’ is short for ‘average’, ‘Sd.’ for ‘standardized’, and ‘Sent.’ for ‘sentence’.

As shown in Table 3, the three-word rhyming translation records a substantially higher type-token ratio (STTR = 47) than the annotation (37.05), poem-style (44.00), and interpretive versions (26.95), reflecting greater lexical variety and denser information (Richards, 1987). Its couplets average 6.32 words, slightly above the expected 6.00 from the 3 + 3 source structure. This difference is mainly due to contractions (e.g., do not, what’s, you are), which are counted as two words by AntConc but function as single prosodic units to preserve rhyme and parallelism. Most distinctively, the rhyming version’s average sentence length (6.32) is less than half that of the other varieties (15.47, 13.63, and 15.24, respectively), highlighting condensed syntax achieved by omitting articles and auxiliary verbs. Such compression may theoretically reduce parsing demands and provide more regular chunking cues, though cognitive load and memory performance need to be directly measured in future research.

Figure 3 shows the entropy of the four text varieties for words of all numbers of letters.

**Figure 3:**
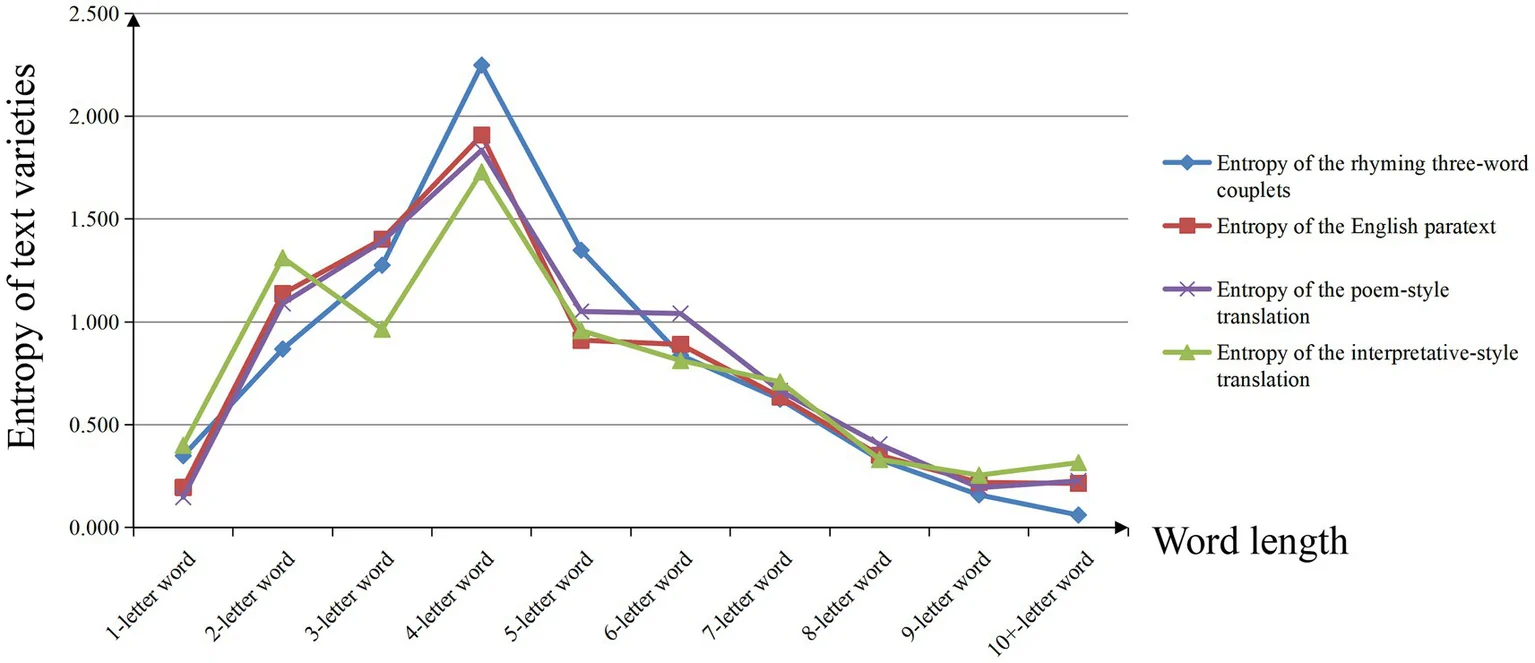
Entropy of the four text varieties (Source: created by the authors).

Overall, the four versions show a similar trend: entropy rises from 1-letter words, peaks at 4-letter words, and then declines through 10-letter words. The rhyming translation exhibits a high entropy value (*H* = 8.10), closely approximating that of the ST (*H* = 8.30) (see Table 4) and notably higher than the annotation (*H* = 7.86) and interpretive versions (*H* = 7.82), indicating relatively high lexical information dispersion despite prosodic compression. Additionally, the rhyming translation is characterized by a predominance of four-letter words, which account for 26.89% of its total tokens. Such monosyllabic items facilitate rhythmic and metrical parallelism, a defining formal property of this rhyming-couplet style.

**Table 4:** RR, *P1*, *Pcum10*, *H*, and lexical uniqueness.

Text	RR	*P_1_*	*Pcum10*	*H*	Lexical uniqueness
Chinese ST	0.0061	3.98	16.94	8.30	80.10%
Three-word rhyming translation	0.0097	5.98	23.64	8.10	84.26%
English annotation	0.0144	7.94	28.94	7.86	77.38%
Poem-style translation	0.0128	6.85	28.54	8.04	80.90%
Interpretive translation	0.0159	7.47	31.86	7.82	76.94%

Table 4 reports repetition rate (RR), *P₁, Pcum₁₀*, entropy (*H*), and lexical uniqueness for all text varieties.

As shown in Table 4, lexical uniqueness peaks in the rhyming translation (84.26%), confirming a strategy of diverse lexical selection. The ST shows the lowest RR, *P₁*, and *Pcum₁₀*, and the highest entropy (*H* = 8.30). Notably, its entropy is closer to that of the rhyming version (*H* = 8.10) than to the other translations (*H* = 8.04 and 7.82), indicating a level of lexical information dispersion closer to that of the ST. Moreover, the higher entropy of the rhyming and poem-style versions (8.10 and 8.04 vs. 7.86 and 7.82) suggests richer and less predictable lexical patterning, which may contribute to aesthetic appeal rather than directly determine aesthetic quality. Such an interpretation is consistent with accounts of literary experience in which aesthetic processing is responsive to formal textual properties (Jacobs, 2015), as well as empirical evidence associating lexical variation and novelty with aesthetic evaluation in poetry and language more generally (Simonton, 1990; McGregor et al., 2019). In contrast, annotative and interpretive styles rely on more formulaic clarity with limited vocabulary.

Collectively, these quantitative indicators reveal a clear gradient of stylistic variation across the four text varieties. The three-word rhyming translation is characterized by low lexical repetition, low high-frequency word concentration, high information density, and high lexical uniqueness. In contrast, the annotation and interpretive translations exhibit the opposite pattern: higher formulaic repetition, greater function-word dominance, and lower information density. The poem-style translation occupies an intermediate position, balancing formal poetic constraints with explanatory clarity.

#### 3.1.3 Cross-version lexical and phraseological patterns

These stylistic patterns are further manifested in the distribution of high-frequency lexical items and recurrent phraseological structures, as shown in Tables 5, 6. Table 5 presents the top 10 high-frequency words for each corpus, while Table 6 lists the top 10 two-word lexical bundles—units that capture conventionalized usage patterns and reveal systematic differences in translator style. For comparability across corpora of unequal sizes, frequency data in Tables 5, 6 were normalized to occurrences per 1,000 words. The normalized patterns corroborated the raw-frequency findings reported below.

**Table 5:** Top 10 high-frequency words of the four varieties.

Rank	Three-word rhyming translation (raw/per 1,000 words)	English annotation	Poem-style translation	Interpretive translation
1	you (68/59.75)	you (231/79.41)	you (171/68.54)	I (213/74.74)
2	the (36/31.63)	should (108/37.13)	and (83/33.27)	will (133/46.67)
3	a (32/28.12)	your (88/30.25)	your (83/33.27)	and (101/35.44)
4	your (25/21.97)	and (84/28.88)	to (76/30.46)	my (90/31.58)
5	what (19/16.7)	if (64/22)	the (61/24.45)	to (80/28.07)
6	no (18/15.82)	to (63/21.66)	should (55/22.04)	not (70/24.56)
7	do (17/14.94)	when (57/19.59)	be (49/19.64)	the (70/24.56)
8	same (16/14.06)	a (50/17.19)	not (49/19.64)	if (59/20.7)
9	if (14/12.3)	the (50/17.19)	in (44/17.64)	a (48/16.84)
10	good (13/11.42)	not (47/16.16)	if (41/16.43)	is (44/15.44)

Raw frequencies are shown before the slash; normalized frequencies (per 1,000 words) are shown after the slash.

**Table 6:** Top 10 high-frequency two-word lexical bundles of the four varieties.

Rank	Three-word rhyming translation	English annotation	Poem-style translation	Interpretive translation
1	What’s (11/9.67)	you should (85/29.22)	you should (37/14.83)	I will (106/37.19)
2	Do not (10/8.79)	if you (38/13.06)	if you (21/8.42)	If I (32/11.23)
3	do not (7/6.15)	when you (29/9.97)	do not (18/7.21)	will not (32/11.23)
4	you are (7/6.15)	do not (23/7.91)	when you (15/6.01)	my parents (26/9.12)
5	if they (4/3.51)	you are (21/7.22)	your parents (15/6.01)	I am (21/7.37)
6	if you (4/3.51)	it is (13/4.47)	should be (14/5.61)	I must (17/5.96)
7	the same (4/3.51)	should be (13/4.47)	should not (12/4.81)	when I (17/5.96)
8	Who’s (4/3.51)	your parents (13/4.47)	you are (8/3.21)	it is (16/5.61)
9	you do (4/3.51)	You have (11/3.78)	you will (8/3.21)	an elder (10/3.51)
10	a good (3/2.64)	You can (10/3.44)	will be (7/2.81)	to be (10/3.51)

Raw frequencies are shown before the slash; normalized frequencies (per 1,000 words) are shown after the slash.

A comparative analysis of the top 10 high-frequency words (Table 5) and two-word bundles (Table 6) reveals significant stylistic divergences, most pronounced between the three-word rhyming translation and the other versions. The rhyming translation achieves lexical richness through content-word density (e.g., good, same, no) to sustain rhyme, while minimizing functional words (e.g., should). This pattern may have cognitive implications: greater content-word density concentrates lexical information, while the reduced presence of function words may encourage a more chunk-based parsing strategy. These possibilities remain theoretical interpretations rather than directly measured processing effects. By contrast, the annotation relies on fixed syntactic patterns (e.g., it is, your parents, you should) favouring clarity over creativity. The poem-style version offers a middle ground, balancing form and grammar, whereas the interpretive version is notably subjective and explicit, marked by frequent first-person forms (I = 213/74.74, my = 90/31.58, I will = 106/37.19) that replace the rhyming version’s impersonal you with semantic precision.

Overall, the rhyming translation prioritizes rhythmic innovation over grammatical rigidity, while the other versions emphasize functional or explanatory clarity. Its frequent use of “you,” roughly twice as common as other terms, reinforces its vocative nature through parallelism and rhythmic repetition, a prosodic design that is consistent with theoretical accounts linking rhythmic regularity to chunking and reduced processing demands (Miller, 1956; Thalmann et al., 2019).

#### 3.1.4 Syntactic patterns of the three-word rhyming translation

Based on the two-word lexical bundles of *CFD* (Table 6), we list the most used sentence patterns of the three-word rhyming translation in Table 1.

As shown in Table 1, the rhyming couplets abound in imperative sentences, conditional clauses, noun phrases, and patterns such as simple present tense and modal verbs, expressing admonishment, persuasion, resolve, and introspection.

This instructional text is characterized by a highly constrained syntactic repertoire—imperatives, conditionals, and elliptical noun phrases—which are not merely stylistic choices but defining features that support its communicative function. Strict grammatical completeness is subordinated to phonological conciseness and rhythmic symmetry. The most frequent structure—the positive imperative—appears twice as often as the next most common forms, delivering direct instruction or advice. Related patterns, including “You + verb + object” construction and negative imperatives, serve similar functions, with occasional inversion to accommodate rhyme. A further notable feature is the elliptical, title-like noun phrase (e.g., A great aim, a good name), which becomes meaningful through its accompanying annotation.

Across stanzas, parallelism, repetitive couplets, and trisyllabic rhyme coalesce to produce an inscription-like textual format, one that yields chanted verses whose musical rhythm heightens emotional resonance. Phonological compactness and rhythmic symmetry are prioritized over strict grammatical completeness. Through the strategic interplay of imperatives, conditional expressions, and consistent second-person address, the translation highlights its vocative orientation: it seeks to capture attention, establish immediacy, and engage readers affectively.

### 3.2 Zipf distribution patterns and lexical concentration (RQ2)

This section examines lexical distribution properties across translations. The slope of a text’s Zipf distribution provides an indicator of lexical concentration and diversity, with differences in the slope reflecting variation in lexical richness and stylistic organization (Koplenig, 2018).

Figure 4 shows the Zipf distributions of the original Chinese verses (Figure 4a), three-word rhyming couplets (Figure 4b), annotation (Figure 4c), interpretive translation (Figure 4d), and poem-style translation (Figure 4e) of *Dizi Gui*. Each point in these sub-figures represents a word’s frequency rank (*x*-axis) against its actual frequency (*y*-axis).

**Figure 4:**
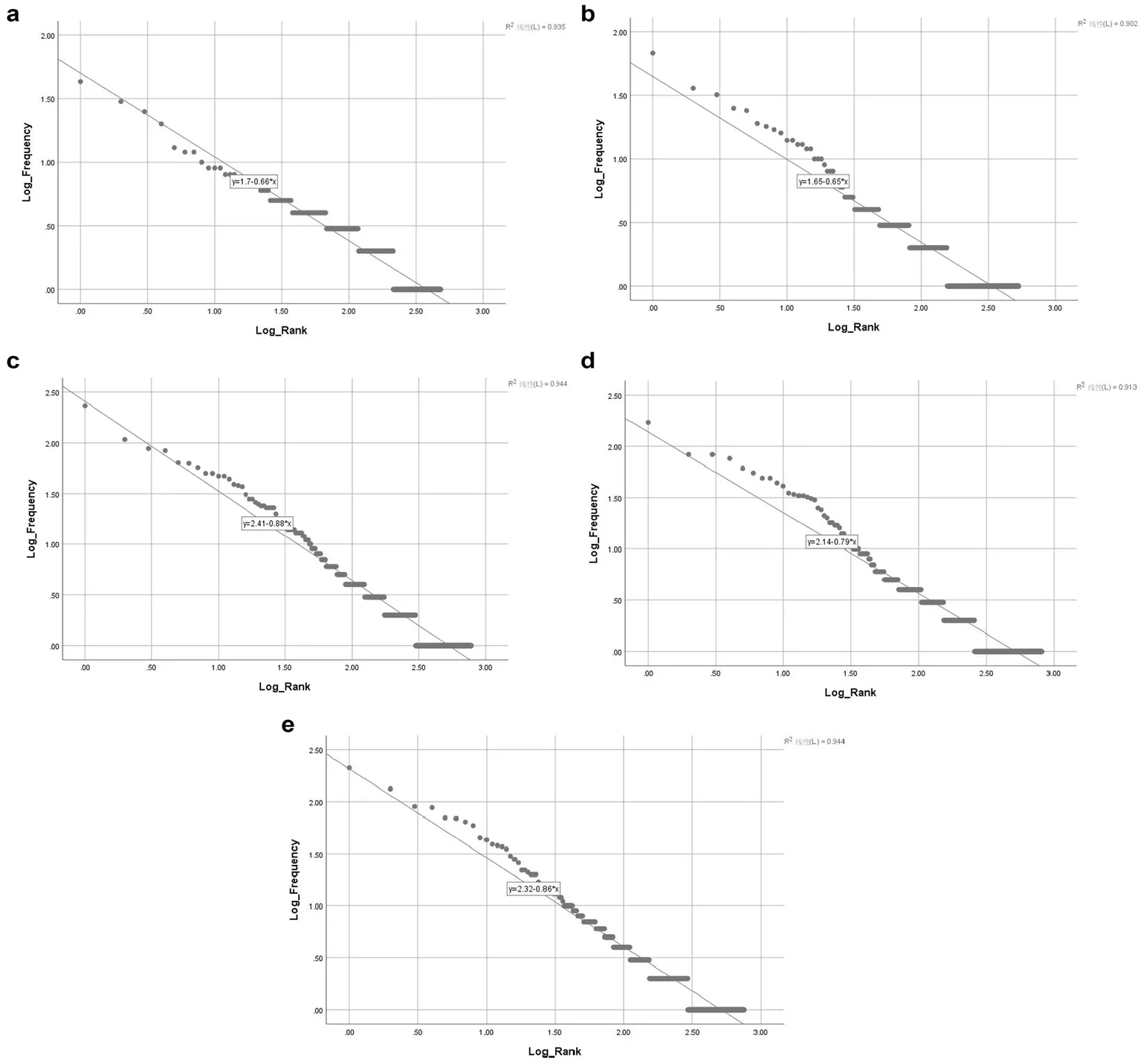
Zipf distributions of the ST and the selected translations: **(a)** original Chinese verses (ST); **(b)** three-word rhyming translation; **(c)** English annotation; **(d)** poem-style translation; and **(e)** interpretative translation. Source: created by the authors.

The annotation (*β* = −0.88) and interpretive translation (*β* = −0.86) show steeper slopes, indicating greater lexical concentration and repetition. The three-word rhyming translation (*β* = −0.65) exhibits a slope closely approximating that of the ST (*β* = −0.66), indicating a similar macro-level pattern of lexical concentration. As a theoretical hypothesis, rather than a direct inference from the Zipfian measure, this distributional alignment may hypothetically reduce cross-linguistic prediction errors, potentially allowing readers to allocate fewer attentional resources to lexical decoding relative to other translations. The resulting processing fluency could, in principle, leave more attentional capacity available for deeper semantic integration and memory encoding. This hypothesized processing mechanism may help account for the higher perceived mnemonic effectiveness of the three-word rhyming translation, though these cognitive consequences were not directly measured in the present study.

### 3.3 Reader reception of the three translation styles (RQ3)

To examine reader reception of this three-word rhyming translation style, Table 7 summarizes student participants’ perceived preferences for the three translation styles across perceived mnemonic effectiveness, aesthetic appeal, and popularization potential. A series of chi-square goodness-of-fit tests revealed that reader preferences significantly deviated from an equal distribution across the three translation styles for all three evaluative dimensions.

**Table 7:** Reader reception of the three translation styles across three evaluation dimensions (*N* = 115).

Translation style	Highest perceived mnemonic effectiveness	Highest perceived aesthetic appeal	Highest perceived popularization potential
*CFD*	74 (64.35%)	49 (42.61%)	66 (57.39%)
The interpretive translation	23 (20%)	26 (22.61%)	18 (15.65%)
The poem-style translation	18 (15.65%)	40 (34.78%)	31 (26.96%)

For perceived mnemonic effectiveness, the distribution of responses differed significantly from chance, *χ*^2^(2) = 50.10, *p* < 0.001, *V* = 0.47, indicating a medium-to-large effect. *CFD* was selected by nearly two-thirds of respondents, substantially exceeding both the interpretive translation and the poem-style translation. Bonferroni-adjusted pairwise comparisons confirmed that *CFD* was preferred significantly more often than both the interpretive translation (adjusted *p* < 0.001, Cohen’s *h* = 0.94) and the poem-style translation (adjusted *p* < 0.001, Cohen’s *h* = 1.06), both indicating large effects. No significant difference was observed between the interpretive and poem-style translations (adjusted *p* = 0.434, Cohen’s *h* = 0.11).

For perceived aesthetic appeal, the overall distribution was also significant, *χ*^2^(2) = 7.01, *p* = 0.030, *V* = 0.17, indicating a small effect. The *CFD* version received the highest proportion of selections, followed by the poem-style translation and the interpretive translation. Bonferroni-adjusted pairwise comparisons showed that *CFD* was preferred significantly more often than the interpretive translation (adjusted *p* = 0.008, Cohen’s *h* = 0.43). No significant differences were found between the rhyming and poem-style translations (adjusted *p* = 0.340, Cohen’s *h* = 0.16) or between the interpretive and poem-style translations (adjusted *p* = 0.085, Cohen’s *h* = 0.27).

For perceived popularization potential, a significant overall preference pattern was again observed, *χ*^2^(2) = 32.16, *p* < 0.001, *V* = 0.37, indicating a medium effect. The *CFD* version was chosen by 57.39% of respondents, compared with 26.96% for the poem-style translation and 15.65% for the interpretive translation. Bonferroni-adjusted pairwise comparisons confirmed a significant preference for *CFD* over both the interpretive translation (adjusted *p* < 0.001, Cohen’s *h* = 0.90) and the poem-style translation (adjusted *p* < 0.001, Cohen’s *h* = 0.63), indicating large to medium-to-large effects. No significant difference was detected between the interpretive and poem-style translations (adjusted *p* = 0.063, Cohen’s *h* = 0.28).

Collectively, *CFD* received the highest proportion of selections across all three dimensions, with the strongest advantage observed for perceived mnemonic effectiveness. This perceptual pattern is consistent with theoretical accounts of prosodic processing, although the contribution of the supplementary annotation cannot be isolated in the present design. The lexical prosody of the ST, characterized predominantly by monosyllabic words, provides a phonological foundation for chunking. Within the *CFD* condition, the primary three-word TT, characterized by the rigid three-word line structure, regular end-rhyme, and syntactic parallelism, may reduce processing demands by offering predictable structural cues, which may support working-memory processing by providing potential retrieval cues (Thalmann et al., 2019).

In contrast, the interpretive translation introduces high structural variability, which may increase processing demands by requiring readers to expend additional resources identifying sentence boundaries. The poem-style translation, while retaining some rhythmic elements, lacks the strict prosodic constraints of the three-word version, leading to less consistent chunking cues. Because highly regular sound sequences have been associated with more automated decoding, such regularity may allow fewer attentional resources to linguistic parsing and more to information retention (Blain et al., 2022). This proposed mechanism may help account for the stronger perceived mnemonic preference for the *CFD* condition, particularly given that the three-word rhyming text served as the primary reading material; such processing savings, if present, could potentially support memory consolidation (Gkintoni et al., 2024).

## 4 Case illustrations (RQ3)

To illustrate how prosodic regularity operates at the phrase and clause level, this section presents two representative examples from the three-word rhyming translation. The first (Table 8) illustrates the ‘aabb’ rhyme scheme with assonance, and the second (Table 9) illustrates perfect end rhyme with syntactic ellipsis—together capturing the two most common prosodic devices employed across the 360 couplets. Specifically, these cases illustrate textual features that may theoretically contribute to reduced cognitive load and support working memory consolidation.

**Table 8:** Example 1.

Text type	Content
ST	泛爱众, 而亲仁。 有余力, 则学文。
Three-word rhyming translation	The masses above, Everyone you love. With more energy, Further your study.
Annotation	As a member of the society, you should love others, and learn from those with good virtues. If you have more than enough time and energy, you should carry on with your study.
Poem-style translation	Befriend all the people around you, But stay close to those with virtue. If you have done these and have time to spare, For search for knowledge you must care.
Interpretive translation	Furthermore, it teaches us to love all equally, and to be close to and learn from people of virtue and compassion. Only when we have accomplished all the above can we then study further and learn literature and art to improve the quality of our cultural and spiritual lives.

**Table 9:** Example 2.

Text type	Content
ST	凡是人, 皆须爱。 天同覆, 地同载。
Three-word rhyming translation	Love every one Under the sun. The same Nature, The same creature.
Annotation	All men, regardless of creed, race or religion, are of the same kind, and should love each other. Created by the same Nature, they should sustain this community through close cooperation.
Poem-style translation	All human beings yearn for love, Despite their differences in status; We all live under the same sky above, And on the same earth supporting us.
Interpretive translation	Human beings, regardless of nationality, race, or religion—everyone—should be loved equally. We are all sheltered by the same sky and we all live on the same planet Earth.

### 4.1 Prosodic compression paired with annotation for semantic clarification

Table 8 shows a typical example of how intense prosodic compression can be systematically coupled with paratextual annotation to potentially reduce processing demands and preserve core semantic content.

To accommodate cross-linguistic variations, the three-word rhyming translation adopts an “aabb” rhyme scheme that mirrors the structural conciseness of the ST. Specifically, the three English words in the first and second lines utilize morphologically and phonologically compressed items to directly correspond to the trisyllabic clausal template of the ST. By leveraging a liberal rendering, the final words “above” and “love” share the identical trailing phonemes (/ʌv/), establishing a perfect rhyme (Knoop et al., 2021). Conversely, at the interface of the third and fourth lines, “energy” and “study” achieve a subtle assonance (Cushman et al., 2012). This acoustic pattern is deliberately engineered through the strategic omission of the ST’s explicit reference to “文” (literary study). In the original philological context, “学文” narrow-mindedly denotes “studying literature” but broadly signifies “acquiring knowledge.” By relegating this broad conceptual expansion to the paratextual annotation, the verse is freed to prioritize phonological optimization without introducing semantic distortion or reader disorientation. Within this formal framework, the assonance between trailing syllables successfully counterbalances the lack of overt grammatical connectives, ensuring that the TT’s meter and rhythmic cadence remain structurally identical to the ST when intoned.

From a psycholinguistic perspective, this precise formal organization may have cognitive implications. While alternative varieties rely on informative verbosity or introduce high structural variability, the three-word rhyming translation strictly preserves a minimalist architectural format characterized by strict end-rhyme and rigid syntactic parallelism. Through radical syntactic ellipsis, the translator systematically purges determiners, auxiliaries, and explicit logical connectives from the clausal stream. This extreme syntactic compression may minimize extraneous cognitive load by stripping the text of non-essential function words (Ekin et al., 2025), while the highly predictable, regular auditory–visual sequences may facilitate automated decoding (Blain et al., 2022; Gkintoni et al., 2024). Consequently, if such processing savings occur, attentional resources may become more available for memory consolidation, potentially allowing readers to group the sparse linguistic input into rhythmically coherent cognitive chunks—a form of organization that has been associated with mnemonic support in previous research (Miller, 1956; Thalmann et al., 2019).

In contrast, the alternative text varieties demonstrate how the absence of rigorous prosodic constraints may increase processing demands. By expanding the concise original verse into explicit grammatical prose, the interpretive-style translation introduces severe explanatory redundancy. Formulations such as “Furthermore, it teaches us to..” and “Only when we have accomplished all the above..” rely heavily on formulaic, non-essential function words, potentially increasing demands on the reader’s visual processing channel without comparable prosodic cues that might support memory encoding. Similarly, while the poem-style translation achieves some aesthetic appeal, its loose metrical structures and shifting sentence lengths (“Befriend all the people..” vs. “If you’ve done these and have time..”) introduce structural unpredictability. Without fixed syllabic boundaries, readers may need to allocate greater attentional resources to parse fluctuating clause structures, which could interfere with automated processing and potentially weaken working memory consolidation (Baddeley, 2012; Ekin et al., 2025). Finally, the prose annotation, serving exclusively as an explicit semantic commentary, completely discards phonological patterning, highlighting the functional contrast between the explicit semantic support of the annotation and the phonological patterning of the three-word variant.

Another example (Table 9) is as follows:

The three-word translation is an example of perfect end rhyme, with two/ʌn/s and /tʃə(r)/s. To achieve this, the translator freely renders “凡是” (all) into “under the sun”, preserving its core meaning. Syntactically, using ellipsis through omission of connectives and verbs in the second sentence, the translator crafts a poetic line that is readily understandable, potentially memorable, and grammatically acceptable in a colloquial vocative text. Logically, readers may not immediately grasp the underlying logic and meaning. This minor ambiguity in meaning, however, can be easily clarified through annotation. While the poem-style translation achieves rhythmic beauty through rhyming and conveys emotional resonance, its semantic accuracy is slightly compromised by poetic adjustments and narrowed contextual scope. By contrast, the three-word rhyming translation exhibits features associated with aesthetic appeal and perceived mnemonic effectiveness. Furthermore, the inclusion of annotations serves to clarify its meaning. Interpretive translation excels in logical clarity and semantic completeness by directly elaborating on the original text’s universal values, though it lacks the linguistic artistry and phonological patterning of rhymed versions.

In sum, Chinese classical canonical works with unique textual structures can be rendered in highly divergent styles, though *CFD*, with its primary three-word TT preserving the original classical format, received the highest proportion of selections in each of the three evaluated dimensions.

### 4.2 Thematic fidelity under prosodic constraint

The above two examples demonstrate how this three-word rhyming translation integrates prosodically optimized verse with semantically explicit annotation—a dual-mode strategy that distinguishes it from both the poem-style and interpretive translations.

To examine whether prosodic compression affects thematic content, we identified the most frequent content words in each version. In the three-word rhyming translation, the top content words include no, good, best, kind, elder, care, study, heart, love, and piety. This set overlaps substantially with the high-frequency content words of the ST and its annotation (e.g., good, care, respect, love, study), indicating that prosodic compression does not necessarily entail thematic distortion. Despite its formal economy, the rhyming couplets preserve the ST’s core philosophical lexicon. By contrast, the poem-style translation shifts toward general action verbs (make, take, say), and the interpretive version emphasizes first-person pronouns (I, my, we). Contrary to the intuition that prosodic constraints necessarily reduce semantic richness, these lexical differences suggest that the three-word rhyming translation, through its formal constraints, may provide stronger potential retrieval cues for memory while showing substantial lexical preservation compared to function-word-dominant alternatives.

## 5 Discussion

### 5.1 Prosodic regularity and cognitive processing

This study investigated how prosodic regularity may influence cognitive processing in visually presented three-word rhyming couplets. Using a four-pronged framework combining corpus analysis, Zipfian distribution modeling, qualitative illustration, and reader perception data (*N* = 115), we compared four English varieties of a classical Chinese canonical text. The three-word rhyming couplets—characterized by fixed three-word lines in couplet form, regular end-rhyme, and syntactic parallelism—showed extreme syntactic compression, high lexical diversity (TTR = 46.67%), and a Zipfian slope (−0.65) nearly identical to that of the ST (−0.66). Reader evaluations showed that this highly regular prosodic variety received the highest proportion of selections across all three measured dimensions, although its advantage over the poem-style translation in perceived aesthetic appeal was not statistically significant.

These patterns are consistent with established principles of human information processing (Miller, 1956; Baddeley, 2012). Information, as Chunking theory and working memory models posit, can be grouped into meaningful, predictable units (chunks) to optimize limited cognitive capacity (Thalmann et al., 2019). Prosodic predictability, as confirmed by recent psycholinguistic studies (LaCroix and Ratiu, 2025; O’Leary et al., 2025), further reduces cognitive load during sentence processing. The three-word rhyming translation is consistent with these proposed mechanisms by utilizing fixed three-word couplets and regular end-rhyme, potentially allowing readers to group words into rhythmically coherent chunks with reduced parsing effort. Crucially, the observed extreme syntactic compression—achieved by omitting determiners, auxiliaries, and explicit connectives—effectively eliminates non-essential function words, which may reduce extraneous cognitive load (Ekin et al., 2025) and may leave more attentional resources available for memory consolidation. This raises the possibility that, rather than challenging the syntax-first psycholinguistic assumption that syntactic category processing is a necessary prerequisite for semantic integration (Friederici, 2002, 2011; Bornkessel-Schlesewsky and Schlesewsky, 2008), highly regular prosodic organization may compensate, at least partially, for reduced grammatical explicitness. The present design, however, does not directly test the temporal relationship between syntactic and semantic processing, and this possibility therefore requires direct experimental investigation.

The syntactic and prosodic optimization in our study has a clear computational basis in the text’s micro-level lexical and macro-level structural features. The TT’s average word length (4.13 letters) aligns precisely with that of highly memorable English poems (Forsyth, 2000), which are optimized for rapid visual parsing and vocabulary simplification. Furthermore, its high entropy value (*H* = 8.10) exceeds that of standard Modern English prose (4.03) and comparable poetic texts (O’Keeffe and Rundell, 1989; Kozhemyakina et al., 2023), indicating relatively high lexical information dispersion, while its effect on psychological processing effort remains to be directly tested. This structural economy, as further illuminated by Zipfian distribution analysis, demonstrates that the rhyming translation closely mirrors the ST’s lexical concentration. Such distributional alignment indicates that intense formal compression can coexist with a macro-level lexical concentration pattern closely resembling that of the ST. Whether this similarity has consequences for semantic processing or memory remains a hypothesis rather than a direct inference from the Zipfian analysis. Because the text varieties differ in total word count, however, the normalized high-frequency-word and lexical-bundle patterns should still be interpreted with some caution. Although normalization substantially improves comparability across unequal corpora, frequency estimates for less common items may remain more sensitive to sampling variation in shorter texts, particularly in phraseological analysis (Hyland and Jiang, 2018; Wolfer and Koplenig, 2025). Accordingly, these normalized frequency patterns are treated here as converging evidence for the broader lexical and structural tendencies identified across the analyses, rather than as corpus-size-independent effects in their own right.

### 5.2 Implications for form-preserving translation

Beyond these phonological features and possible cognitive implications, this study bridges a persistent gap between cognitive science and translation studies by providing reader-reception evidence relevant to the potential functional value of form preservation. For decades, cross-cultural translation scholars have debated whether preserving the formal features of canonical texts enhances or hinders communication (Nida, 1964; Chesterman, 2004). Stylistic features of STs can be systematically reproduced in TTs while maintaining comparable functional effects, as shown by recent corpus-based research (Ryu et al., 2023). These textual alignments, while suggestive, do not address the critical question of whether such formal reproduction translates to favorable reader perceptions of mnemonic effectiveness, aesthetic appeal, and popularization potential. Our questionnaire data extend this line of inquiry by showing that, in the present comparison, the *CFD* condition, which combined prosodically regular verse with supplementary annotation, received the highest selection proportion in each of the three evaluative dimensions, although its aesthetic advantage over the poem-style translation was not statistically significant.

Such a format represents a promising strategy for translating ancient canonical works. The three-word rhyming translation demonstrates that rigid prosodic constraints can be successfully adapted across typologically distinct linguistic systems to create a new, formally optimized text genre. For example, adapting Shakespearean sonnets into five-character or seven-character Chinese verse forms may represent a parallel application of these cognitive principles, suggesting that preserving the ST’s rhythmic structure may offer a useful direction for examining cross-cultural reader reception of form-preserving translations. Such intentional stylistic choices, framed by the concept of creative fidelity (Chesterman, 2004), bridge academic rigor and public accessibility, potentially supporting perceived mnemonic effectiveness and emotional engagement while preserving the unique identity of the ST.

### 5.3 Limitations and future directions

Despite these merits, this study has several limitations. First, and most importantly, the post-task questionnaire assessed each of the three reception-oriented dimensions using a single forced-choice item. Although this format was designed to capture relative preferences among competing translation varieties, it does not measure the intensity of individual judgments or permit assessment of internal-consistency reliability. Accordingly, the questionnaire findings should be interpreted primarily as relative perceived preferences rather than comprehensive measurements of the evaluative dimensions. In particular, perceived mnemonic effectiveness should not be equated with actual memory performance. Future studies should therefore supplement forced-choice comparisons with multi-item subjective rating scales and direct behavioral measures, such as free recall and recognition tests. Validated mental-effort ratings, eye-tracking (Hvelplund, 2017), or EEG (Liu et al., 2025) could further provide more direct evidence concerning the proposed cognitive-load and processing mechanisms.

Second, the study focuses exclusively on *Dizi Gui*. While this allows in-depth analysis, it restricts the generalizability of findings to other related genres (e.g., lyric poetry, historical records) or non-Chinese texts (e.g., Western sonnets, Arabic proverbs). Future research may extend the four-pronged methodology to other formally constrained and mnemonic-oriented traditions, such as *The Three Character Classic*, which shares with *Dizi Gui* a trisyllabic instructional structure rooted in oral transmission (Kern, 2010), Japanese haiku, which relies on extreme brevity and compression to achieve aesthetic and cognitive effects (Shirane, 1998), and Persian ghazals, which are characterized by highly regular rhyme and prosodic organization (Meisami, 1987). Applying the framework across these traditions would help evaluate the generalizability of the patterns observed among prosodic regularity, lexical information dispersion, and reader-reception measures.

Third, the questionnaire participants were translation students recruited from a single institution rather than lay readers representative of the broader target audience. Their systematic training in translation and familiarity with classical Chinese textual conventions may have made them more sensitive to rhyme, structural correspondence, and formal fidelity. In addition, because individual familiarity with *Dizi Gui* was not measured, pre-existing knowledge of the ST may have reduced semantic processing demands and potentially influenced participants’ evaluations of the different translation styles. Although all translation conditions were based on the same ST, this design cannot rule out an interaction between prior knowledge and translation style. Consequently, the observed preference for the three-word rhyming translation may have been amplified by their disciplinary background and may not fully reflect the responses of general readers, who might place greater emphasis on semantic explicitness and immediate comprehensibility. Future research should replicate the study with more diverse samples, particularly non-translation students and lay readers from different linguistic and educational backgrounds, while explicitly assessing or controlling participants’ prior familiarity with the ST.

An additional limitation concerns the composition of the reader-comparison conditions. In the *CFD* condition, the three-word rhyming text served as the primary reading material, while its prose annotation was available as a supplementary semantic aid when clarification was needed. The poem-style and interpretive conditions did not include an equivalent supplementary component. Consequently, the observed preference for *CFD* cannot be attributed uniquely to prosodic regularity, because the annotation may have compensated for semantic ambiguity arising from the compressed rhyming form. Future studies should isolate these effects by comparing prosodically regular and less regular texts under equivalent annotation conditions.

## 6 Conclusion

This study investigated how prosodic regularity may influence cognitive processing in visually presented three-word rhyming couplets, using a four-pronged converging framework combining corpus analysis, Zipfian distribution modeling, qualitative illustration, and reader perception data.

The core findings reveal that combining highly regular prosodic structure with supplementary annotation received the highest proportion of selections across all three perceived evaluative dimensions, although its advantage over the poem-style translation in perceived aesthetic appeal was not statistically significant. Notably, extreme syntactic compression achieved through the omission of non-essential function words coexists with a macro-level lexical concentration pattern closely resembling that of the ST while potentially providing mnemonic retrieval cues. These results suggest that rigorous formal regularity may compensate for severe syntactic ellipsis in vocative genres designed for memorization.

Theoretically, this study provides findings consistent with cognitive load theory and chunking theory in visually presented prosodically constrained language, while raising the possibility that prosodic organization may partly compensate for reduced grammatical explicitness, a possibility that requires direct experimental testing. Methodologically, it introduces an integrated four-pronged framework that addresses the longstanding limitation of traditional corpus stylistics, where textual metrics are routinely analyzed in isolation from human psychological reception. This framework successfully triangulates objective textual features with subjective reader experiences, providing a more comprehensive understanding of the relationship between text structure and reader perception. Practically, these findings provide implications for the translation and dissemination of classical canonical texts. The dual-mode design combining a prosodically optimized core verse with explicit prose annotations offers a promising strategy for balancing formal economy, perceived mnemonic effectiveness, and semantic clarity.

## Data Availability

The datasets of the source corpora and questionnaire are publicly available at https://doi.org/10.5281/zenodo.17233084.

## Footnotes

1
https://www.calculator.net/sample-size-calculator.html
